# A self-stabilized coherent phonon source driven by optical forces

**DOI:** 10.1038/srep15733

**Published:** 2015-10-27

**Authors:** D. Navarro-Urrios, N. E. Capuj, J. Gomis-Bresco, F. Alzina, A. Pitanti, A. Griol, A. Martínez, C. M. Sotomayor Torres

**Affiliations:** 1 Catalan Institute of Nanoscience and Nanotechnology ICN2, Bellaterra (Barcelona), Spain; 2 NEST, Istituto Nanoscienze – CNR and Scuola Normale Superiore, Piazza San Silvestro 12, Pisa, I-56127; 3 Depto. Física, Universidad de La Laguna, La Laguna, Spain; 4 Instituto Universitario de Materiales y Nanotecnología, Universidad de La Laguna, La Laguna, Spain; 5 Nanophotonics Technology Center, Universitat Politècnica de València, Spain; 6 Catalan Institute for Research and Advances Studies ICREA, Barcelona, Spain

## Abstract

We report a novel injection scheme that allows for “phonon lasing” in a one-dimensional opto-mechanical photonic crystal, in a sideband unresolved regime and with cooperativity values as low as 10^−2^. It extracts energy from a cw infrared laser source and is based on the triggering of a thermo-optical/free-carrier-dispersion self-pulsing limit-cycle, which anharmonically modulates the radiation pressure force. The large amplitude of the coherent mechanical motion acts as a feedback that stabilizes and entrains the self-pulsing oscillations to simple fractions of the mechanical frequency. A manifold of frequency-entrained regions with two different mechanical modes (at 54 and 122 MHz) are observed as a result of the wide tuneability of the natural frequency of the self-pulsing. The system operates at ambient conditions of pressure and temperature in a silicon platform, which enables its exploitation in sensing, intra-chip metrology or time-keeping applications.

Coherent creation, manipulation and detection are key ingredients for the full technological exploitation of particle excitations. While lasers and masers have dominated among the sources in electromagnetism, the natural lack of discrete phonon transitions in solids make the realization of coherent vibration sources a formidable challenge. The introduction of miniaturized self-sustained coherent phonon sources is crucial in applications such as mass-force sensing^1^ and intra-chip metrology and time-keeping^2^. They also offer intriguing opportunities for studying and exploiting synchronization phenomena among several oscillators by introducing a weak interlink^3^,^4^, which can be optical^5^,^6^ or mechanical^7^.

In recent years several versions of “phonon lasers” have been reported in electro- and opto-mechanical systems^1^,^8^,^9^,^10^. The most common optical pumping mechanism is by means of the retarded radiation pressure within a nano/microcavity through a sideband-assisted energy scattering process^11^. The stringent requirements to reach the lasing threshold, e.g., cooperativity values close to unity^12^, restricts its effective operation to high quality factor modes with large opto-mechanical (OM) coupling strengths. Alternatively, an external feedback can be used to parametrically increase the amplitude of the mechanical oscillations up to the lasing regime, but the technological requirements of electronic circuitry limits the maximum mechanical frequency operation^13^,^14^.

In this work we present an opto-mechanical integrated coherent phonon source that realizes “phonon lasing” with relaxed requirements for both the optical and mechanical modes, and their inter-coupling strength. It derives from a spontaneously triggered thermal/free carrier self-pulsing and the anharmonic modulation of the radiation pressure force that comes as a consequence. Moreover, the feedback of the mechanics on the self-pulsing frequency-entrains both, creating a self-stabilized indecomposable system.

The device presented here^15^ is a one dimensional OM photonic crystal fabricated using standard silicon (Si) nanofabrication tools (*see* Supplementary Discussion 1). As seen from the SEM top-view of Fig. 1A, the crystal lattice constant is quadratically reduced towards the center of the beam, hereby defining optical cavity modes.

Low-power, optical transmission measurements of the second order even-even optical mode (see Finite-Element-Method (FEM) simulation in inset of Fig. 1B), show a Lorentzian lineshape with a cavity resonant wavelength at *λ*_*r*_ = *λ*_*o*_ = 1527.01 nm and a linewidth Δ*λ*_*o*_ = 70 pm, holding an optical Q-factor 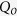 = 2.2 × 10^4^ (Fig. 1B). The same measurement at high input powers shows the typical bistability “saw-tooth” shaped transmission, caused by the red-shift of the resonance frequency due to thermo-optic (TO) nonlinearities^16^. In this regime, decreasing the laser-cavity detuning from the blue-side results in an increase of the intracavity photon number (*n*_*o*_).

Thermally driven motion of the low frequency mechanical modes is seen by processing the transmitted light with a spectrum analyzer. Here, mechanical modes with a non-negligible OM coupling rate (*g*_*OM*_) appear as narrow Lorentzian peaks in the frequency spectrum, as reported in Fig. 1C. The odd, in-plane flexural modes of the beam have a single particle coupling rate (*g*_*o,OM*_) of tens of KHz, as obtained from FEM simulations in a perturbative approach for shifting material boundaries^17^. The even-mode family is weakly coupled due to symmetry considerations. In this work, we focus on the second (three anti-nodes) and third (five anti-nodes) order odd modes, which have a center frequency of ν_*m,*2_ = 54 and ν_*m,*3_ = 122 MHz, simulated effective masses *m*_*eff,*2_ = 2.4 × 10^−15^ kg and *m*_*eff,*3_ = 2.6 × 10^−15^ kg, and mechanical Q-factors *Q*_*m,*2_ = 450 and *Q*_*m,*3_ = 520, respectively. The Q-factors are mainly limited by the experimental conditions: room-temperature (thermo-elastic damping) and atmospheric pressure.

Temporally resolving the optical transmission with a fast detector reveals a complicated nonlinear dynamics when *n*_*o*_ is above *n*_*o,th*_ = 4.4 × 10^4^ (black curve in Fig. 2A). It is well known that the dynamic competition among different nonlinearities can lead to instabilities, self-sustained oscillations and chaotic behavior of light within a photonic device^18^,^19^,^20^. The two main sources of nonlinearities in standard Si resonators operating at 1.55 μm are Free-Carrier Dispersion (FCD) and the TO effect^16^. In the case of FCD, the excess of free-carriers leads to a reduction of the material refractive index and therefore a blue-shift of the optical cavity mode^21^. On the other hand, the TO effect results in an increase of the refractive index of the material with increasing temperature. Here other high-order effects, such as Kerr nonlinearity, are ignored^16^. Since the main source of heating is Free-Carrier Absorption (FCA), the dynamics of free-carrier density (*N*) and the temperature increase (Δ*T)* are linked and can be described by a system of coupled rate equations^22^,^23^:


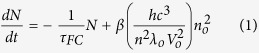



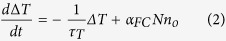


where *h* is the Planck constant, *c* is the speed of light, *V*_*o*_ is the optical mode volume and *α*_*FC*_ is defined as the rate of temperature increase per photon and unit free-carrier density. In the FCD (Eq. 1) we consider a Two-Photon Absorption (TPA) generation term^23^, where *β* is the tabulated TPA coefficient^16^ and a surface recombination term governed by a characteristic lifetime τ_*FC*_. The TO effect (Eq. 2) reflects the balance between the fraction of photons that are absorbed and transformed into heat due to FCA and the heat dissipated to the surroundings of the cavity volume, which is governed by a characteristic lifetime τ_*T*_. The generation terms of Eqs 1 and 2 depend on *n*_*o*_, which includes the cavity filtering effect:


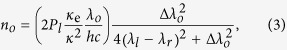


where the term in parentheses represents the maximum *n*_*o*_ achieved during the oscillation, κ_*e*_ and κ are the extrinsic and total optical decay rates respectively, *λ*_*l*_ is the laser wavelength and *P*_*l*_ is the input power (we have assumed that *λ*_*r*_ ∼ *λ*_*o*_ for the maximum *n*_*o*_ factor and that Δ*λ*_*r*_*∼*Δ*λ*_*o.*_). Importantly, 
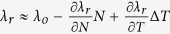
 is the cavity resonant wavelength including first order nonlinear effects. Note that in Eqs 1 and 2 we have neglected spatial temperature gradients (∂Δ*T*/∂*x*, ∂Δ*T*/∂*x* = 0) and assumed a homogenous distribution of *N*. Moreover, we consider an adiabatic response of the optical mode to the refractive index change: this comes naturally when we take into account the very different time-scale of the FCD and TO effects with respect to the optical cavity decay of ∼20 ps.

For particular sets of conditions, *N* and Δ*T* form a stable limit cycle (*see* Supplementary Discussion 4), known as self-pulsing (SP)^22^. This phenomenon has been observed in various photonic structures, such as microdisks and photonic crystals^22^,^23^,^24^,^25^,^26^. In our system, when *n*_*o*_is above *n*_*o,th*_, the system enters in the SP regime and the transmission assumes an asymmetric, double dip shape coming from the interplay between fast (FCD) and slow (TO) dynamics. Numerical integration of Eq. 1 reproduces accurately the experimental data, as seen in Fig. 2A. The characteristic lifetimes giving the best agreement with the experimental data are: τ_*T*_ ∼ 0.5 *μ*s and τ_*FC*_ ∼ 0.5 ns, which are compatible with values reported elsewhere^23^. Interesting insights can be gained from the inspection of the 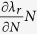
(FCD) and 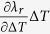 (TO) simulated time traces of Fig. 2C. Within the limit cycle the temperature is unable to reach the steady state, which reflects in the triangular waveform of Fig. 2C (red curve). The slow temperature decay (mirrored to a wavelength increase in the panel) dominates the SP oscillating frequency (ν_*SP*_), being the free-carrier creation and recombination very fast at these time scales. Since the Δ*T* instantaneous decay rate depends on the absolute value of the temperature, ν_*SP*_ can be enhanced up to five times by increasing the amount of total heat in the cavity. In our system this is simply done by increasing *λ*_*l*_, which increases the time-averaged *n*_*o*_. In order to further increase its operation regime, a reasonable strategy is to speed up the thermal/free-carrier dynamics by increasing the thermal diffusivity of the OM structures and the free-carrier recombination rate.

When the SP is active, the light within the cavity is modulated in a strongly anharmonic way, creating an “optical comb” in the frequency domain with multiple peaks spectrally located at integers of ν_*SP*_ (*see* Supplementary Discussion 5). This results in a modulation of the radiation pressure optical force (*F*_*o*_), which can be easily evaluated as a function of the intracavity photon number since 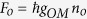. The flexural modes of the nanobeam can be described as damped linear harmonic oscillators driven by the anhamonic force:


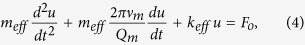


where *u* is the generalized coordinate for the displacement of the mechanical mode and *k*_*eff*_ is its effective spring constant. Finally, the nonlinear wavelength has to include now the effect of the mechanical motion when evaluating Eq. 3, as:


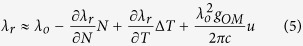


Importantly, the response of *n*_*o*_ to deformation is also adiabatic since the radiative lifetime of the optical mode (∼20 ps) is three orders of magnitude smaller than the mechanical oscillation period (∼ 20 ns).

The dynamics of the two systems described by Eqs 1, 2 and 4 are coupled through *n*_*o*_. The most significant feature of the coupled system is that self-sustained mechanical motion is achieved if one of the low harmonics of the SP main peak at ν_*SP*_ is resonant with the mechanical oscillations (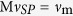, where *M* is an integer number) (*see* Supplementary Discussion 5). In fact, the coherence of the mechanical oscillation is maintained since the mechanical mode lifetime (∼10 *μ*s) is much larger than 1/*M*ν_*SP*_ (∼20 ns). As an example, Fig. 2B shows experimental and theoretical data for the case corresponding to *M* = 2 and the 3^rd^ in-plane flexural mode. As expected, the mechanical oscillation at frequency ν_*m*_ is superimposed on a SP trace at frequency ν_*m*_*/2*. Separating all the terms contributing to *λ*_*r*_ (Fig. 2D), we see that a simple harmonic signal (blue curve denoted by OM) is included with the FCD and TO signal to obtain the data of Fig. 2B. Our model reproduces quite well the dynamics of the transmitted signal, revealing that the contribution to *λ*_*r*_ of the displacement *u* is of similar magnitude of the ones from *N* and Δ*T*. The coherent oscillation of the mechanics implies that the driving strength overcomes the mechanical dissipation in the system, entering a regime that has been defined as “phonon lasing”^2^, although the particle generation mechanism is very different to the one in photon systems. In this regime, phonons are created coherently, as seen from the self-sustained oscillations of Fig. 2B. It is worth noting that the same “phonon lasing” regime can be achieved through dynamical back-action processes, where for compensating intrinsic mechanical losses is desirable i) to be in the sideband resolved regime (κ/2π <ν_*m*_) and is required ii) to reach a field-enhanced cooperativity, defined as 
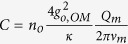
, at least of unity^12^. The SP pumping mechanism reported here is intrinsically different and achieves “phonon lasing” in more relaxed conditions, i.e.: i) while being deeply in the unresolved regime (κ/2π >>ν_*m*_) and ii) having *C* values within the 10^−2^ range.

The full RF power spectrum as a function of *λ*_*l*_presents several regions and features of interest. As shown in Fig. 3A, below threshold (left of the dashed vertical green line) only peaks related to the transduction of the mechanical modes appear. Above threshold, the signature of SP emerges. When the mechanics/self-pulsing resonant condition is achieved, “flat regions” appear, indicating the coherent vibration of the OM photonic crystal. Since all the dynamics is coupled together, the OM oscillations provide an active feedback that stabilizes the SP. In those specific conditions, the two oscillators are frequency-entrained (FE) in a way that the SP adapts its oscillating frequency to be a simple fraction of the mechanical eigenfrequency. Similarly to the case of synchronized oscillators, the lowest *M* values have the largest FE zones^3^. Inspecting the spectra extracted along the white vertical lines of Fig. 3A and reported in Fig. 3B, it is clear that the linewidth in the FE regions (III—black curve) is orders of magnitude narrower than both the SP oscillation (II–red curve) and the mechanical thermal Brownian motion (I–green curve), as expected from a coherent source. Following the SP main frequency peak evolution, we can extract a curve that resembles the Devil’s staircase of synchronized systems^3^, as reported in Fig. 3C (black curve). Several regions of FE appear both with the 2^nd^ and 3^rd^ in-plane flexural odd modes (*M* = 1, 2, …, 6 and *M* = 3, 4 respectively), where ν_*SP*_ is stable (see Supplementary Videos S1 and S2) and robust to wide variations of *λ*_*l*_ and/or the laser power. By tuning *λ*_*l*_ it is possible to sweep over different FE states and switch to frequency-unlocked situations.

These experimental features can be well reproduced (green curve of Fig. 3C) by introducing a *g*_*o,OM*_in good agreement with the evaluated one reported in Fig. 1C. As expected, if the active feedback is turned off (i.e., *g*_*o,OM*_ = 0), all the FE regions disappear and the SP naturally evolves with no interaction with the mechanics. The differences between model and experiment with regard to the spectral width of plateaus with *M* > 1 is associated to the non-linearity of the elastic restoring force and the damping term^27^.

The mechanism of generation of coherent mechanical oscillations shown in this work, with its self-stabilized nature, could represent a viable solution to generate and feed phonons to waveguides or membranes. The relatively low standards of the required technology could lead to a change of paradigm in the emerging field of phononics, by making coherent phonon generation readily available to researchers who have not access to state-of-the-art fabrication facilities and experimental setups. Since it operates on a silicon platform at atmospheric conditions it can represent a first building block for the integration of phononic circuits with electronics and photonics, allowing hybrid systems with added functionalities. Among the latter, these mechanical oscillators can be used as ‘clocks’ to provide reference signals. In this context, synchronization phenomena among networks of self-sustained mechanical oscillators could be exploited to extend further the spatial range of a common coherent signal.

## Methods

### OM crystal geometry

The unit-cell is a Si parallelogram containing a cylindrical hole in the central part and two symmetric stubs on the sides. The cavity region consists of 12 central cells in which the pitch (a), the radius of the hole (r) and the stubs length (d) are decreased in a quadratic way towards the center, with a reduction up to 83% of the values at the extremes. At both sides of the defect region a 10 period mirror is included. The nominal geometrical values of the cells of the mirror are a = 500 nm, r = 150 nm, and d = 250 nm. The whole device length is about 15μm. The whole beam is free-standing and anchored to the rest of the Si layer from the two extremes, forming a stripe clamped at both ends suitable for optical actuation.

### Measurement

A tapered fiber is placed nearly parallel to the OM crystal in order to excite its localized optical modes. A tunable laser source is coupled to the fiber input and the transmitted signal is detected at the output in two ways. To check for the presence of a radiofrequency (RF) modulation of the transmitted an InGaAs fast photoreceiver with a bandwidth of 12 GHz was used. The RF voltage is connected to the 50 Ohm input impedance of a signal analyzer with a bandwidth of 13.5 GHz. The maximum input power used in this work is 1 mW. The whole setup operates at atmospheric conditions of temperature and pressure. (see Supplementary Dicussion 3 for more details)

## Additional Information

**How to cite this article**: Navarro-Urrios, D. *et al.* A self-stabilized coherent phonon source driven by optical forces. *Sci. Rep.* **5**, 15733; doi: 10.1038/srep15733 (2015).

## Supplementary Material

Supplementary Information

Supplementary Movie S1

Supplementary Movie S2

## Figures and Tables

**Figure 1:**
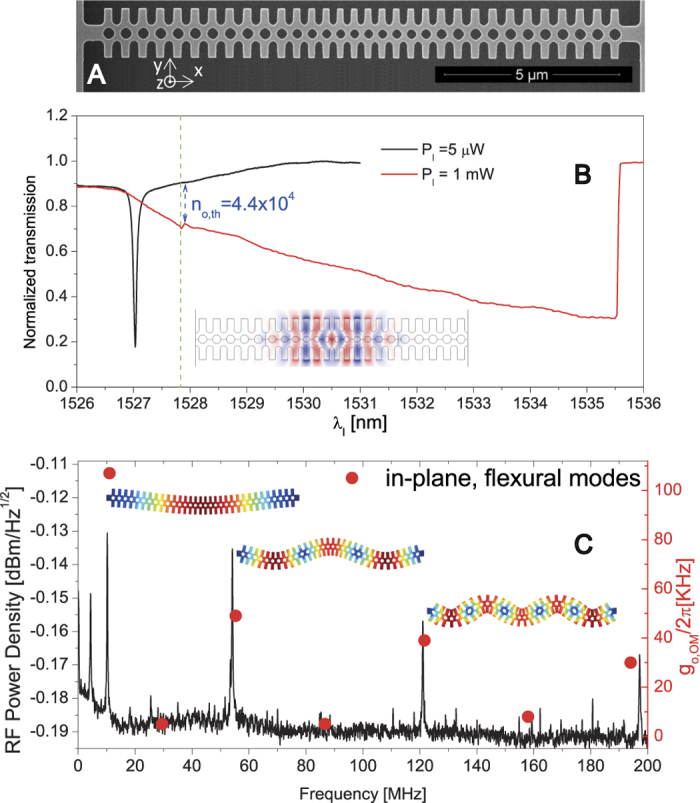
Optical and mechanical responses of the OM photonic crystal. (**A**) SEM micrograph of the OM photonic crystal cavity. (**B**) Normalized transmission spectrum around the studied optical mode for input laser powers of 5 μW and 1 mW (black and red curves respectively). The threshold for establishing the regime of self-sustained oscillation is indicated (green dashed vertical line). (**C**) Transduced acoustic modes up to 200 MHz. The red dots correspond to the *g*_*o,OM*_/2*π* values of the in-plane flexural eigenmodes as predicted by FEM simulations. The OM photonic crystal deformation profiles associated to the first three odd in-plane flexural modes are also illustrated.

**Figure 2:**
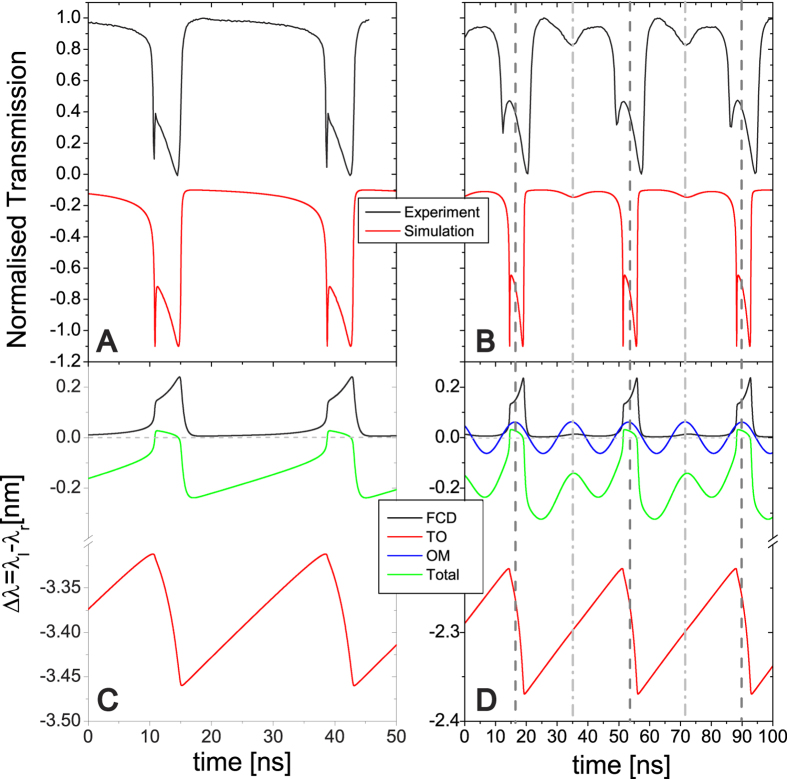
Dynamics of the coupled system for *P*_*in*_ = 1 mW and *n*_*o*_ over the threshold. (**A**,**B**) The black (red) curve shows the experimental (simulated) temporal profile of the transmitted signal obtained at *λ*_*l*_ = 1530.2 nm (panel A) and at *λ*_*l*_ = 1529.1 nm (panel B). In the latter case the OM photonic crystal is oscillating coherently in its 3^rd^ in-plane flexural mode. The SP is frequency-entrained with the mechanical oscillation (*M* = 2) (**C**,**D**) Simulated temporal profiles of FCD (black), TO (red) and OM (blue, only in panel D) contributions to the spectral shift of *λ*_*r*_obtained at *λ*_*l*_ = 1530.2 nm (panel C) and at *λ*_*l*_ = 1529.1 nm (panel D). The overall spectral shift of *λ*_*r*_ with respect to *λ*_*l*_ is represented in green. The dashed horizontal line indicates the resonant condition, i.e., *λ*_*l*_ =  *λ*_*r*._

**Figure 3:**
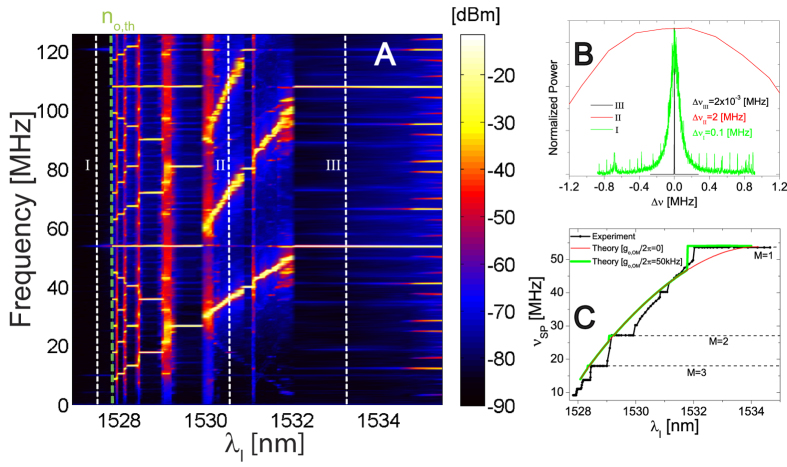
Multiple frequency entrainment and phonon lasing. (**A**) Color contour plot of the RF power obtained for a *P*_*in*_ = 1 mW. The green-dashed line indicates the threshold position. The vertical white-dashed lines indicate situations in which the signal corresponds to: thermally activated motion below threshold (I), SP within a frequency-unlocked regime (II) and frequency-entrained situation for *M* = 1 (III). (**B**) Normalized RF spectra corresponding to the cuts I, II and III of panel A (green, red and black curves respectively), where I illustrates the 2nd in-plane flexural odd mode and II and III the first harmonic of the transduced signal. (**C**) Simulated behavior of ν_*SP*_ considering only the 2^nd^ mode and its estimated value of *g*_*o,OM*_ (green line). The red curve is the expected SP behavior in the absence of feedback, i.e., if the OM photonic crystal were not free to oscillate mechanically or uncoupled with the electromagnetic field. The experimental curve is also included (black dotted line).
