# Supplementary material for: A self-stabilized coherent phonon source driven by optical forces
Source: Sci Rep. 2015 Oct 27;5:15733. doi: 10.1038/srep15733 (PMC4621534; doi:10.1038/srep15733)
Supplement: Supplementary Information [file srep15733-s1.pdf]

Supplementary Information:

Materials and Methods; Figures S1-S11; References (S*1*-*S6*); Videos S1-S2

A self-stabilized coherent phonon-source driven by optical forces

D. Navarro-Urrios[1,2], N. E. Capuj[3,4], J. Gomis-Bresco[1], F. Alzina[1], A. Pitanti[2], A. Griol[5], A. Martínez[5] and C. M. Sotomayor Torres[1,6]

[1] Catalan Institute of Nanoscience and Nanotechnology ICN2, Bellaterra (Barcelona), Spain

[2] NEST, Istituto Nanoscienze – CNR and Scuola Normale Superiore, Piazza San Silvestro 12, Pisa, I-56127

[3] Depto. Física, Universidad de La Laguna, La Laguna, Spain

[4] Instituto Universitario de Materiales y Nanotecnología, Universidad de La Laguna, La Laguna, Spain

[5] Nanophotonics Technology Center, Universitat Politècnica de València, Spain

[6] Catalan Institute for Research and Advances Studies ICREA, Barcelona, Spain

*Correspondence to: daniel.navarrourrios@nano.cnr.it

Materials and methods

S1. Devices

The investigated device is an optomechanical (OM) photonic crystal whose unit-cell contains a hole in the middle and two symmetric stubs on the sides (Fig. S1). The peculiarity of this geometry resides in having a full phononic band-gap at ~4GHz (*1*). The investigation of high frequency mechanical modes is reported elsewhere *(2)*.
We report here the geometrical parameters of our device. In a defect region consisting of 12 central cells the pitch (a), the radius of the hole (r) and the stubs length (d) are decreased in a quadratic way towards the center. The maximum reduction of the parameters is denoted by Γ. At both sides of the defect region a 10 period mirror is included. The nominal geometrical values of the cells of the mirror are a=500nm, r=150nm, and d=250nm. The total number of cells is 32 and the whole device length is about 15μm. Fig. S1 shows a SEM micrograph of one of the OM photonic crystals.
We have fabricated a set of devices in which Γ has been varied from Γ=64% to Γ=83% of the original values. All the results presented in this work correspond to the structure with Γ=83%, but the same effects have been observed for other values of Γ.

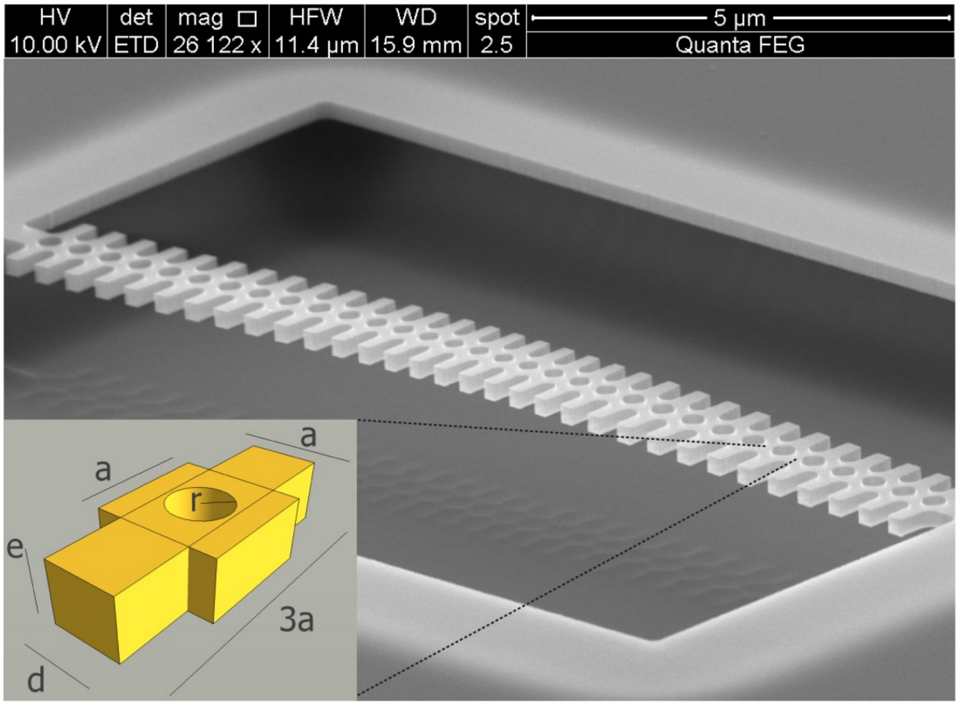

Fig. S1. SEM micrograph of a Γ=83% OM photonic crystal. A sketch of the unit-cell is shown in the inset.

The devices were fabricated in standard silicon-on-insulator (SOI) SOITEC wafers with silicon layer thickness of 220 nm (resistivity ρ ~1-10 Ω cm^{-1}, p-doping of ~10^{15} cm^{-3}) and a buried oxide layer thickness of 2 µm. The pattern was written by electron beam in a 100 nm thick poly-methyl-methacrylate (PMMA) resist film and transferred into silicon by Reactive Ion Etching (RIE). Etching in BHF silicon oxide removed the buried oxide layer and released the beam structures.

S2. Optomechanical coupling calculation

The calculation of $g_{o,OM}$ is performed using the integral given by Johnson et al. (2) for the moving interfaces effects:

$$g_{o,OM} = -\frac{\pi\lambda_o}{c} \oint \boldsymbol{Q} \quad \frac{{}^{2}_{|} - \Delta\varepsilon^{-1} D_{\perp}^{2} \big) dS}{\int E \cdot D dV} \sqrt{h / 2 m_{eff} \nu_m} \qquad \text{(S1)}$$

Where **Q** is the normalized displacement (max{|**Q**|}=1), $\hat{\mathbf{n}}$ is the normal at the boundary (pointing outward), **E** is the electric field and **D** the electric displacement field. ε is the dielectric permittivity, $\Delta\varepsilon=\varepsilon_{silicon}-\varepsilon_{air}$, $\Delta\varepsilon^{-1}=\varepsilon^{-1}_{silicon}-\varepsilon^{-1}_{air}$. λ_o is the cold optical resonance wavelength, c is the speed of light in vacuum, h is the Planck constant, m_{eff} is the effective mass of the mechanical mode and ν_m is the mechanical mode eigenfrequency.

S3. Experimental set-up

The experiments are performed in a standard set-up for characterizing optical and mechanical properties of OM devices.
A tunable infrared laser covering the spectral range between 1460-1580nm is connected to a tapered fiber. The polarization state of the light entering the tapered region is set with a polarization controller. The thinnest part of the tapered fiber is placed parallel to the OM photonic crystal, in contact with an edge of the etched frame (top right photo of Fig. S2a, Fig. S2b). The gap between the fiber and the structure is about 200 nm, as roughly extracted from geometrical considerations using the radius of the fiber loop and the contact point position. A polarization analyzer is placed after the tapered fiber region.
The long tail of the evanescent field and the relatively high spatial resolution (~5 μm^2) of the tapered fiber locally excited the resonant optical modes of the OM photonic crystal. Once in resonance, the mechanical motion activated by the thermal Langevin force causes the transmitted intensity to be modulated around the static value (Fig. S2c).
To check for the presence of a radiofrequency (RF) modulation of the transmitted an InGaAs fast photoreceiver with a bandwidth of 12 GHz was used. The RF voltage is connected to the 50 Ohm input impedance of a signal analyzer with a bandwidth of 13.5 GHz.
All the measurements were performed in an anti-vibration cage at atmospheric conditions of air pressure and temperature.

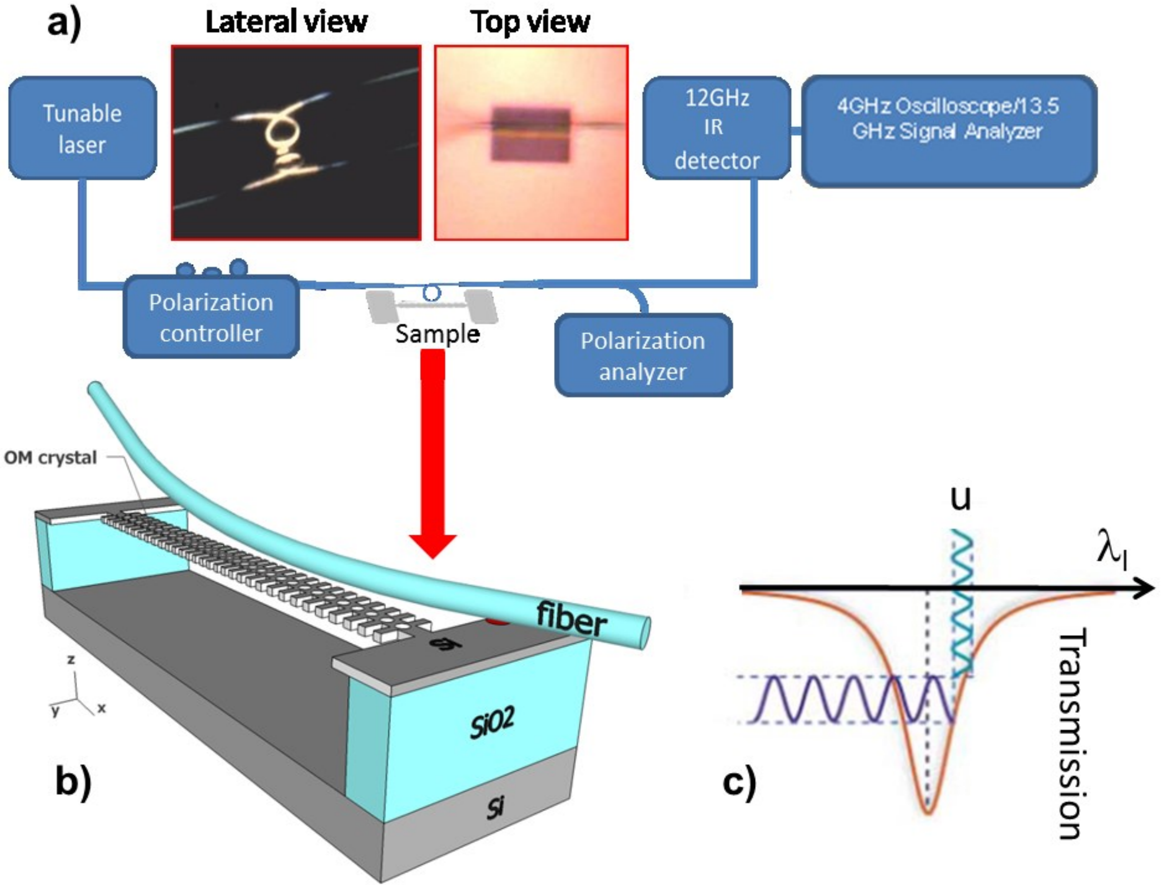

Fig. S2. a). Sketch of the experimental setup to measure the optical and mechanical properties of the OM devices. The sample size has been greatly increased for clarity. The top left photo shows a lateral view of the real microlooped tapered fiber close to the

sample, where the fiber can be seen reflected on the sample. The top right photo shows a top view of the tapered fiber placed parallel with the OM structure and in contact with one of the edges of the etched frame. **b)**. Relative positioning of the tapered fiber and the OM photonic crystal. The leaning point of the fiber is highlighted in red. The fiber is placed close enough to the central part of the OM photonic crystal to excite efficiently its localized photonic modes. **c)** Scheme of the transduction principle.

S3.1. Tapered fiber characteristics and fabrication procedure

The experiments are carried out with tapered optical fibers having diameters in the smallest section of about 1.8μm (Fig. S3a), which is commensurate with the wavelength of interest (around 1.5 μm) and ensures an evanescent field tail of several hundreds of nanometers.
For the fiber fabrication, we used a home-made setup in which a SMF-28 optical fiber is stretched in a controlled way using two motorized stages. The central part of the fiber is placed in a microheater where the temperature is about 1180ºC *(2)*.
The fiber transmission at a wavelength of 1.5 μm is monitored during the pulling procedure (Fig. S3b). The signal is subjected to a short time Fast Fourier Transform(FFT) algorithm, so that the frequency components associated to inference between different supported modes are measured (Fig. S3c). The single mode configuration is achieved when all those frequency components disappear (Fig. S3d).

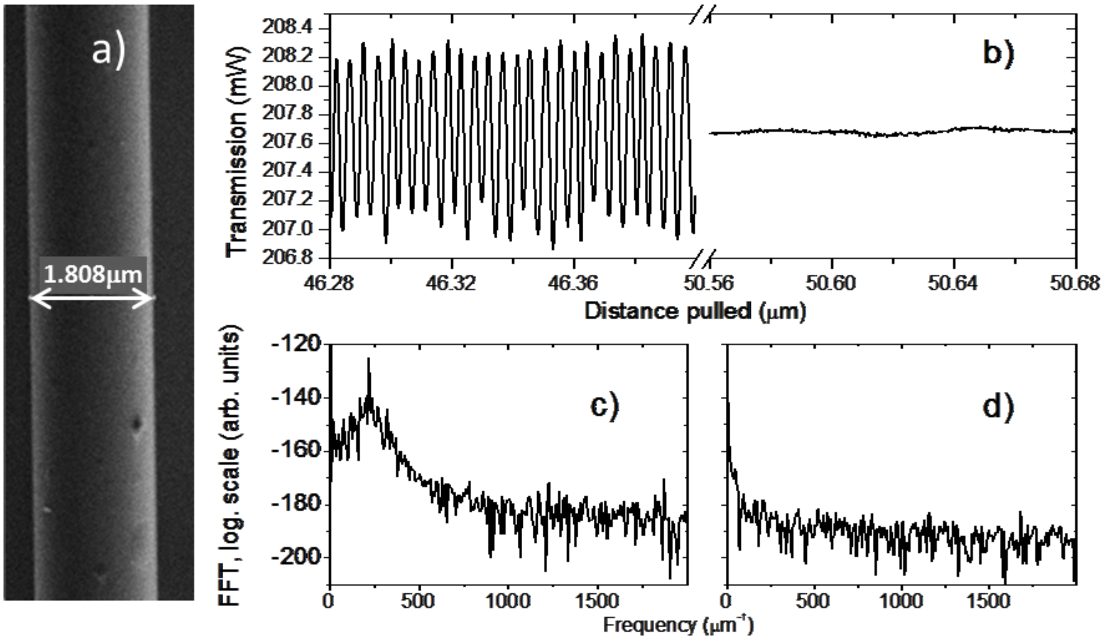

Fig. S3. a) SEM image of the thinnest part of a tapered fiber. **b)** Transition from multimode to monomode while pulling the two extremes of the fiber as seen in temporal scale. **c)** FFT of the transmitted signal before the transition to single mode. **d)** FFT of the transmitted signal after the transition to single mode.

Using two rotating fiber clamps, the tapered fiber is twisted twice around itself. The two fiber ends are gently brought closer over several hundreds of micrometers so that a looped

structure forms in the tapered region. The two fiber ends are afterwards pulled apart to reduce the loop size down to a few tens of μm. In that process, the two parts of the fiber at the loop closing point (upper part of the loop on the top left photo Fig. S2) slide smoothly in opposite senses. The micro-looped shape provides functionalities similar to those of dimpled fibers (*5*).

For the fiber loop photonic structure, the dispersion relation is linear and the group refractive index (n_g) is equivalent to the effective refractive index (n_{eff}). We have calculated n_{eff} using the Beam Propagation Method taking the material refractive index of the cladding of the initial fiber to be n=1.468 at λ_l=1515nm, resulting in a value of n_{eff}=1.373.

S4. Thermo-Optic and /Free Carrier Dispersion induced self-pulsing

S4.1. General behavior.

We have implemented the model first reported by Johnson et al. (6) to describe the dynamics of the self-pulsing (SP) mechanism (Eqs. 1 and 2 of the main text).

The fitting parameters used to reproduce the frequency-unlocked cases are τ_T=0.5 [μs], τ_{FC}=0.5 [ns] and α_{FC}=4x10^{-13} [K cm^3 s^{-1}], while the initial conditions verify that $\frac{\partial\lambda_r}{\partial\Delta T}\Delta T(0)=\lambda_l-\lambda_o$. Thermo-optic (TO) and Free-Carrier-Dispersion (FCD) coefficients were independently calculated by assuming that the observed wavelength shift is only associated to an average change in the Si refractive index within the region overlapping with the electromagnetic fields and using tabulated values for its dependence with temperature and free-carrier density. This procedure lead to the following values: $\frac{\partial\lambda_r}{\partial N}$=7x10^{-19} [nm cm^3] and $\frac{\partial\lambda_r}{\partial\Delta T}$=6x10^{-2} [nm K^{-1}].

Hereafter, we compare the dynamics of the optical transmission, *N*, ΔT and $\Delta\lambda$ in the same time scale (Fig. S4a, panels A, B, C and D respectively). These results are equivalent to those shown in Fig.2C. The SP dynamics is illustrated by the temporal evolution of the resonance-shift (λ_l-λ_r), which contains the overall result of adding the different contributions defining λ_r, i.e., λ_o, FCD and TO effects. An anharmonic behaviour can be observed, with the main oscillation frequency at ν_{SP}. Four specific points of the temporal curves of Fig. S4 are marked with orange numbers:

- On **point 1**, a fast increase of *N* starts due to the absorption of part of the optical energy stored in the cavity.
- The first minimum observed in transmission occurring at **point 2** is related to the quick part of the increase of *N*, which leads to a sign change of λ_l-λ_r from negative to positive values (λ_r blue-shifted with respect to λ_l).
- The second transmission minimum at **point 3** reflects the decrease of N. Again λ_l-λ_r changes sign, this time from positive to negative values (λ_r red-shifted with respect to λ_l). After point 3 the cavity is red-shifted and starts cooling down. In this region N decreases.

- After **point 4**, N stops decreasing and starts a slow increase.

The total time required to complete the $\{\Delta T, N\}$ limit cycle (Fig. S4b) is $1/\nu_{SP}$, although the cycle is not drawn at a constant pace.
A necessary ingredient for the activation of the SP is the time delay of ΔT with respect to N, which can be seen in the temporal traces (see the zoomed panels of Fig. S4a) but is evident in the limit cycle (Fig. S4b). Indeed, the maximum values of N and ΔT are achieved at different positions of the curve.

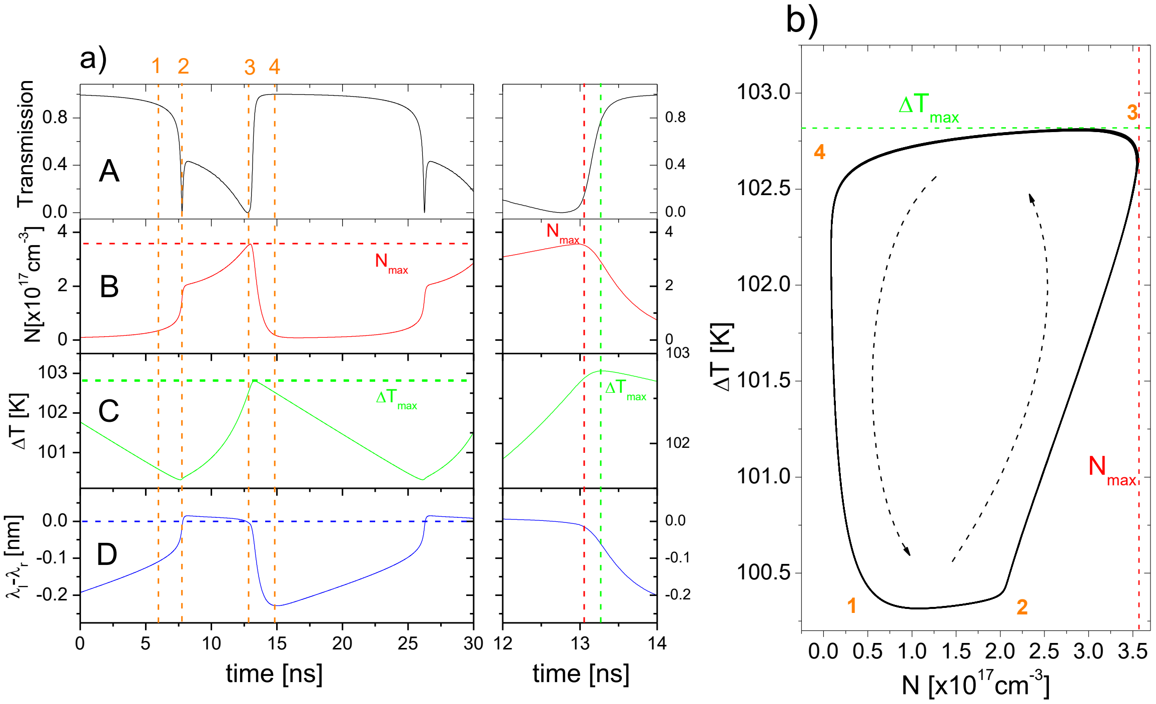

Fig. S4. a) Temporal behaviors of the transmitted optical signal, N, ΔT and λ_l-λ_r (panels A, B, C and D respectively). Specific points in time are indicated with dashed vertical lines. The maximum values of N and ΔT (panels B and C) and the condition $\lambda_l = \lambda_r$ (panel D) are indicated with dashed horizontal lines. A zoomed temporal region is represented in the right panels. **b)** $\{\Delta T, N\}$ limit cycle. The rotation sense is indicated with black dashed arrows. The maximum values of N and ΔT are also indicated with red and green arrows, respectively.

S4.2. Self-pulsing frequency dependence with λ_l

Since the ΔT decay rate depends on its absolute value, ν_{SP} increases with λ_l as the cavity resonance is pushed to longer wavelengths. This is clearly observed when comparing, in the same time scale, the temporal traces of the SP for two well separated values of λ_l (Fig. S5). As expected, the absolute value of the TO contribution (red curves of Fig. S5, note that when the temperature decreases the curves go up) decreases much faster for the longer λ_l. It is also observed that the duty cycle, i.e., the ratio between the time lapse in which TO and FCD are competing and the signal period, also increases with λ_l.

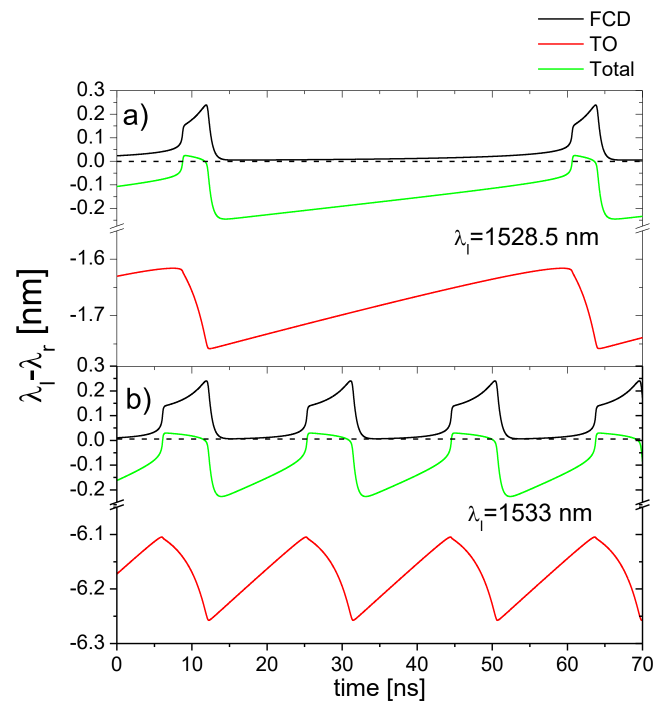

Fig. S5. Simulated temporal profiles of the FCD (black) and TO (red) contributions to the spectral shift of λ_r for λ_l=1528.5 nm (panel **a**) and λ_l=1533 nm (panel **b**). The overall spectral shift is represented in green.

A five-fold enhancement of ν_{SP} from 10 to 50 MHz is shown on Fig. S6, where the FFT of the intracavity optical force is represented. Due to its anharmonicity it is composed by a comb of frequencies, in accordance with the experimental data.

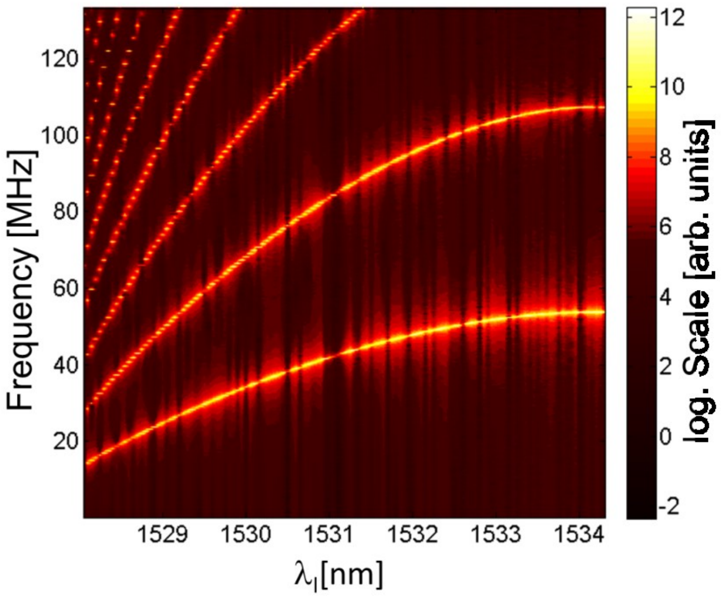

Fig. S6. Color contour plot of the FFT of the intracavity optical force (in log scale).

S5. RF signal and temporal traces dependence with λ_l

S5.1 Coherent mechanical amplification using different harmonics of the force and limit cycles {ΔT, N, u}

When introducing Eqs. (4) and (5) in the model (see main text), the FFT of the optical force as a function of λ_l (Fig. S7a) presents several frequency-entrained (FE) regions at simple fractions of the frequency of the mechanical modes. In those regions the motion of the OM photonic crystal is coherently amplified by one of the harmonics of the force. FigS7b illustrates the former concept by showing the emergence of a peak at ν_m when one of the low harmonics of the force main peak at ν_{SP} is resonant with the mechanics. The stationary solutions for the mechanical displacement are almost pure harmonic signals with small components at the harmonics of ν_{SP}.
In Fig S7 we highlight three situations: I and III cuts fall within FE regions with M=2 and M=1 respectively, II represents a frequency-unlocked region.
Limit cycles are obtained when representing any two variables of the set $\{\Delta T, N, u\}$ versus each other within regions I and III (black and green curves of Fig. S7c). In both previous cases the frequencies of the two oscillators are entrained/locked.
In region II the OM photonic crystal motion cannot be efficiently pumped by any of the harmonics of the force and is forced to oscillate non-resonantly, exhibiting low amplitude and a noisy $\{N, u\}$ or $\{\Delta T, u\}$ limit cycles (red curve of Fig. S7c). A stable $\{\Delta T, N\}$ limit cycle is obtained also in this case.

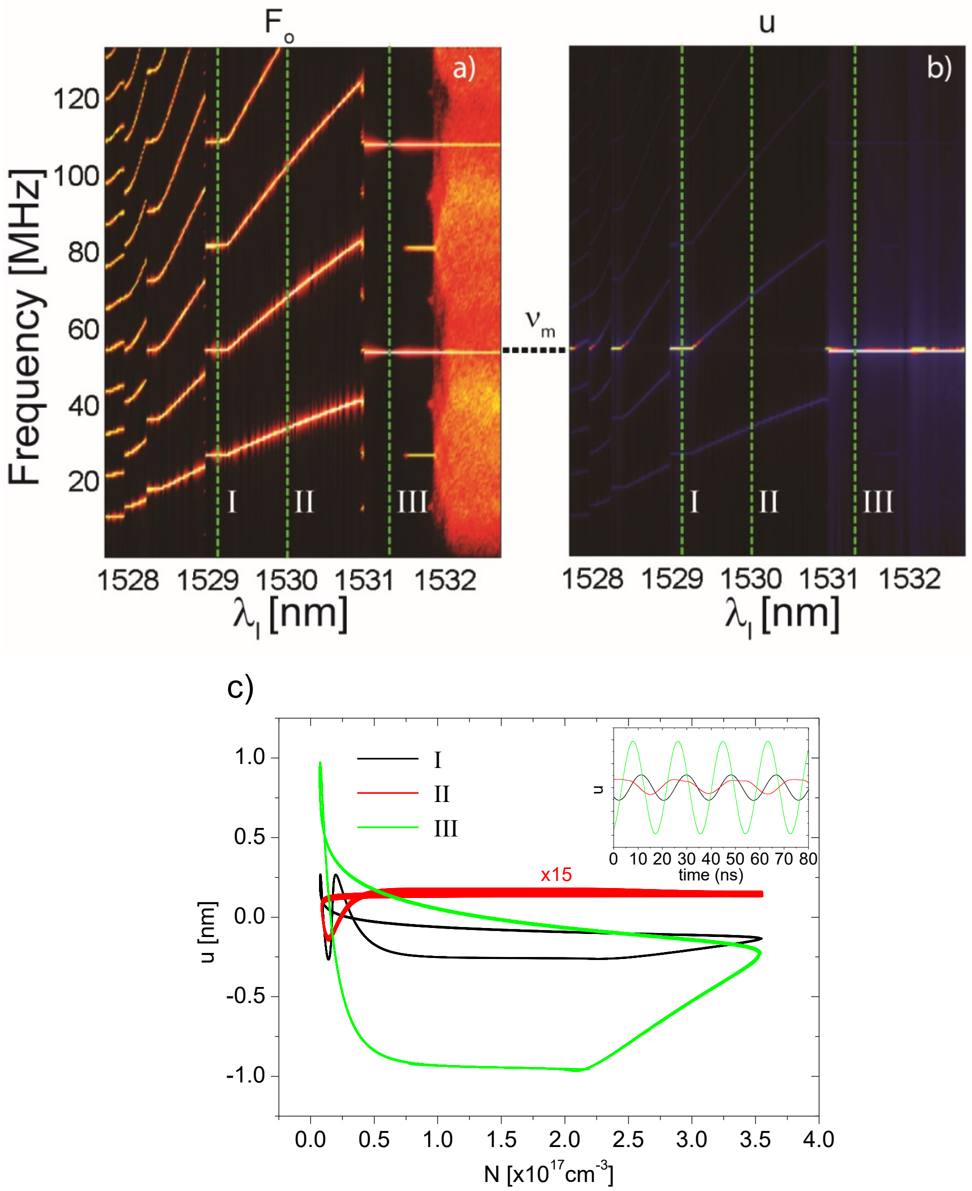

Fig. S7. a) and **b)** Simulated FFT (in log scale) of the optical force (panel a) and *u* (panel b). The vertical cuts correspond to a M=2 and M=1 frequency-locked state (I and III respectively) and to a frequency-unlocked state (II), **c)** {*N, u*} limit cycles on states I, II and III (black, red and green curves respectively). Note that, for the case II, *u* has been enhanced by a factor 15 for comparison. The simulations have been made only considering the 2nd in-plane odd flexural mode (ν_m=54MHz) and its corresponding single-particle OM coupling rate, i.e., $g_{o,OM}/2\pi$=50 kHz.

In the experiment, mechanical modes at frequencies higher than 120 MHz could not be amplified coherently because the strength of the force decays with the harmonic number and cannot compensate the low Q_m values measured at atmospheric conditions. Lower temperatures and pressures of the external environment would increase the Q_m values,

possibly enabling amplification in the GHz range, where the localized modes are present (*2*).

S5.2 Frequency and duty cycle dependence on λ_l within frequency locked and unlocked regions.

In Section 4.2, we showed that the ΔT decay rate increases with λ_l. Within a frequency-unlocked region, the former behavior leads to an increasing of ν_{SP}. On the contrary, within a FE region, the ΔT decay rate increase is compensated by a monotonous increase of the duty cycle (temporal width of the asymmetric transmission minimum), so that ν_{SP} is constant. In Fig. S8a we show the experimental case of M=1, where ν_{SP} locks to ν_m.
This feature is also reproduced by our model when the SP is coupled to the mechanics. In FigS8b we show the simulated temporal traces for two extreme points within the M=1 FE region.

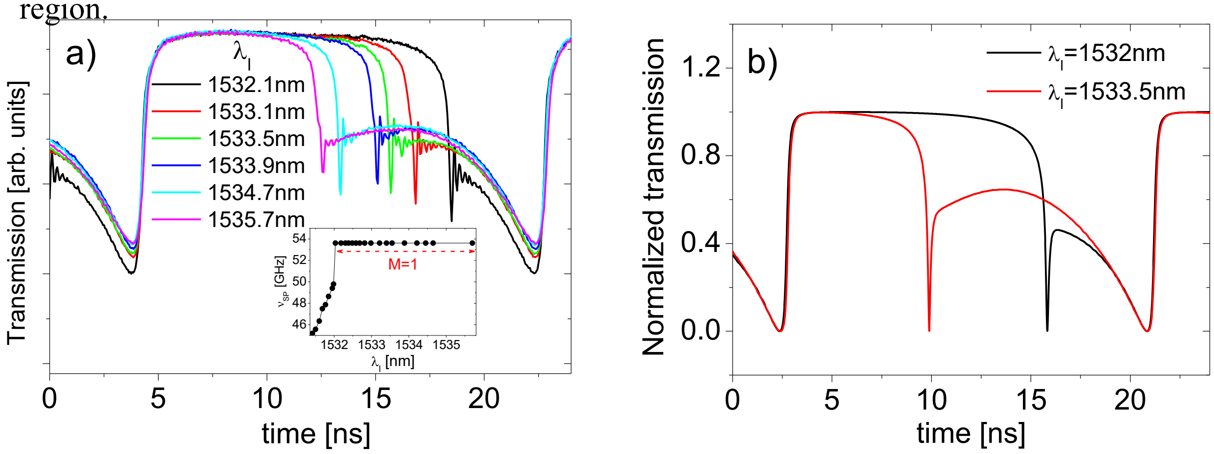

Fig. S8. a) Temporal traces for different values of λ_l within the M=1 FE region. The inset shows the experimental Devil's staircase plot for reference **b)** Simulated transmission for two extreme values of λ_l within the M=1 FE region. The simulations have been made only considering the 2nd in-plane odd flexural mode (ν_m=54MHz) and its corresponding single-particle OM coupling rate, i.e., $g_{o,OM}/2\pi$=50 kHz.

The RF spectra corresponding to extreme situations within the M=1 FE region (Fig. S9) show that the spectral position of the harmonics is obviously the same. However, there is a significant difference on their relative weights, which is consistent with the differences observed in the temporal signals of Fig. S8a.

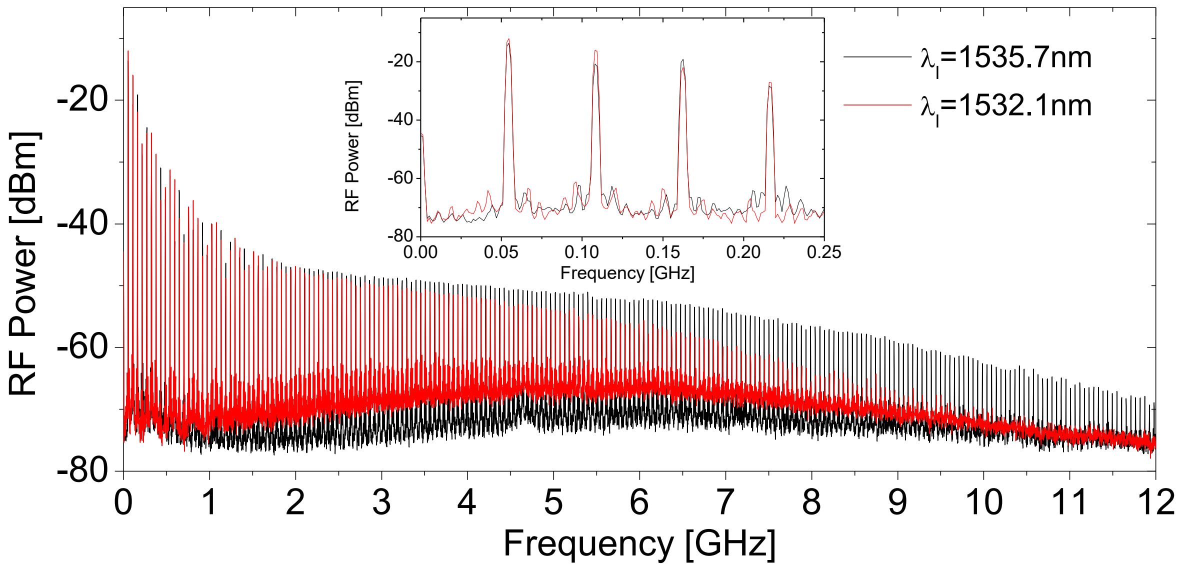

Fig. S9. Frequency combs obtained at the two extremes of the M=1 FE region.

The RF peaks in a frequency-unlocked region (Fig. S10) are inhomogenously broadened in frequency because of the integration time, i.e., the unlocked resonance central frequency oscillates fast in time (less than μs) (Movies S1 and S2). As a consequence, at frequencies above ~1GHz, there is an overlapping of harmonics of different frequencies, which results in a white signal.

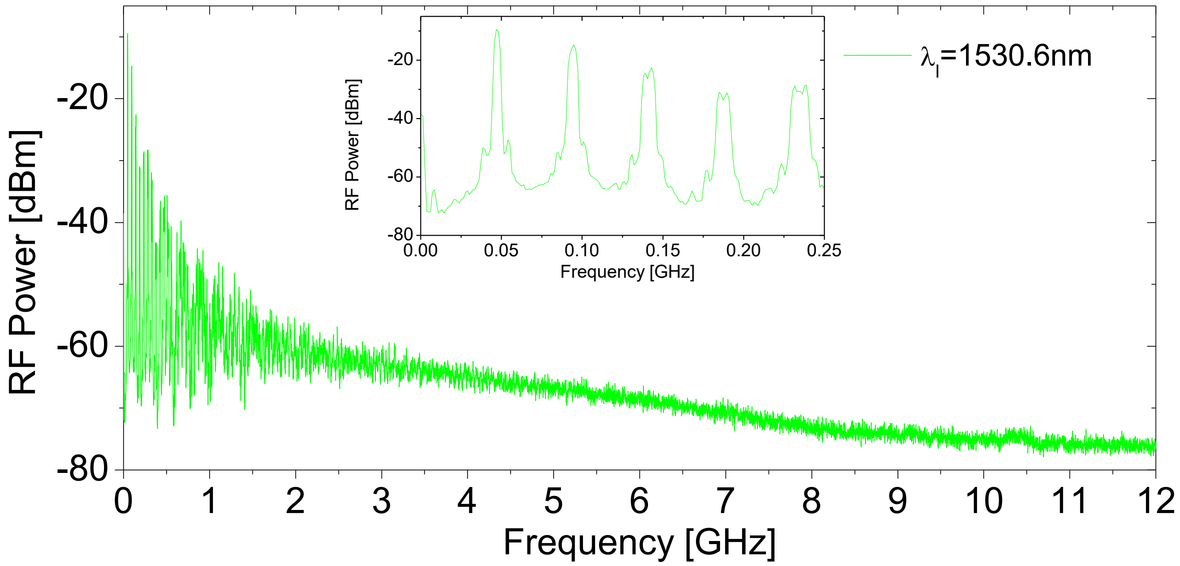

Fig. S10. Frequency comb obtained on a frequency-unlocked region.

S6. Transduction of high frequency mechanical modes when SP is active.

The activation of the SP affects the transduced signal associated to localized mechanical modes in the GHz range. In Fig. S11 we show 2D plot of the RF spectrum of a mode at ν_m=7.3 GHz and its dependence with λ_l. It is worth noting that this particular measurement has been obtained by exciting an optical mode different than the one used throughout the manuscript.

Above the threshold for SP (white dashed line) there is a strong degradation of the transduced signal of one order of magnitude. Moreover, the transduced peak becomes a central peak with equally spaced sidebands that spread apart when λ_l is further increased. This feature is explained in terms of the activation of the SP. In that situation, the thermally activated motion of the localized mode weakly hypermodulates the anharmonic transmitted signal. Consequently, the RF signal associated to the localized mode is no longer a single peak placed at ν_m but a symmetric comb of peaks at $\nu_m \pm M\nu_{SP}$ (M=0, 1, 2, …). It is worth noting that, under the experimental conditions used to obtain the data shown in Fig. S11, the SP is unlocked over the whole range covered by λ_l.

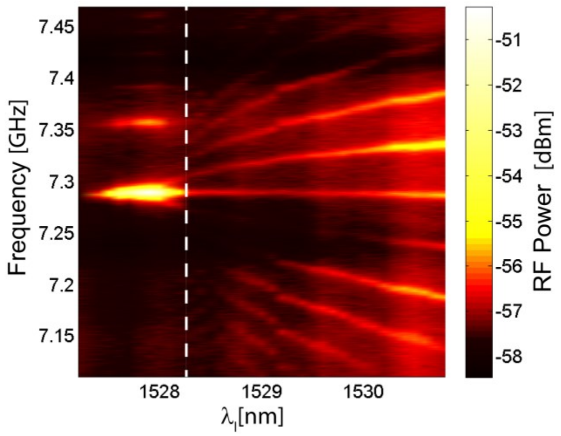

Fig. S11. Color contour plot of the RF power of a localized mechanical mode at ν_m = 7.3 GHz obtained for P_l=0.5 mW. The threshold for the SP activation is indicated with a white vertical line.

References

(1) Y. Pennec et al., *AIP Advances* **1**, 041901 (2011).
(2) J. Gomis-Bresco et al. *Nat. Commun.* **5**, 4452 (2014).
(3) S. G. Johnson et al., *Phys. Rev. E* **65**, 066611(2002).
(4) L. Ding, C. Belacel, S. Ducci, G. Leo, I. Favero, *Appl. Opt.*, **49**, 2441-2445. (2010)
(5) C. P. Michael, M. Borselli, T. J. Johnson, C. Chrystal, O. Painter, *Opt. Express,* **15**, 4745-4752 (2007).
(6) T. J. Johnson, M. Borselli, O. Painter, *Opt. Express*, **14**, 817-831 (2006).

Movies

Video S1: RF signal as a function of laser wavelength with a frequency span of 200MHz

Video S2: RF signal as a function of laser wavelength with a frequency span of 6GHz
